# Obesity is associated with a distinct brain-gut microbiome signature that connects Prevotella and Bacteroides to the brain’s reward center

**DOI:** 10.1080/19490976.2022.2051999

**Published:** 2022-03-20

**Authors:** Tien S. Dong, Michelle Guan, Emeran A. Mayer, Jean Stains, Cathy Liu, Priten Vora, Jonathan P. Jacobs, Venu Lagishetty, Lin Chang, Robert L. Barry, Arpana Gupta

**Affiliations:** a Department of Medicine, Vatche and Tamar Manoukian Division of Digestive Diseases Los Angeles, USA; b Department of Medicine, David Geffen School of Medicine Los Angeles, USA; c Department of Medicine, UCLA Microbiome Center, David Geffen School of Medicine at UCLA Los Angeles, USA; d Department of Medicine, G. Oppenheimer Center for Neurobiology of Stress and Resilience Los Angeles, USA; e Department of Medicine, University of California, Los Angeles, USA; f Division of Gastroenterology, Hepatology and Parenteral Nutrition, VA Greater Los Angeles Healthcare System, Los Angeles, CA, USA; g Department of Radiology, Athinoula A. Martinos Center for Biomedical Imaging, Massachusetts General Hospital, Charlestown, MA, USA; h Department of Radiology, Harvard Medical School, Boston, MA, USA; i Harvard-Massachusetts Institute of Technology Health Sciences & Technology, Cambridge, MA, USA

**Keywords:** Brain-Gut-Microbiome, Prevotella, Bacteroides, Obesity, nucleus accumbens

## Abstract

The prevalence of obesity has risen to its highest values over the last two decades. While many studies have either shown brain or microbiome connections to obesity, few have attempted to analyze the brain-gut-microbiome relationship in a large cohort adjusting for cofounders. Therefore, we aim to explore the connection of the brain-gut-microbiome axis to obesity controlling for such cofounders as sex, race, and diet. Whole brain resting state functional MRI was acquired, and connectivity and brain network properties were calculated. Fecal samples were obtained from 287 obese and non-obese participants (males n = 99, females n = 198) for 16s rRNA profiling and fecal metabolites, along with a validated dietary questionnaire. Obesity was associated with alterations in the brain’s reward network (nucleus accumbens, brainstem). Microbial diversity (p = .03) and composition (p = .03) differed by obesity independent of sex, race, or diet. Obesity was associated with an increase in *Prevotella*/*Bacteroides* (P/B) ratio and a decrease in fecal tryptophan (p = .02). P/B ratio was positively correlated to nucleus accumbens centrality (p = .03) and negatively correlated to fecal tryptophan (p = .004). Being Hispanic, eating a standard American diet, having a high *Prevotella*/*Bacteroides* ratio, and a high nucleus accumbens centrality were all independent risk factors for obesity. There are obesity-related signatures in the BGM-axis independent of sex, race, and diet. Race, diet, P/B ratio and increased nucleus accumbens centrality were independent risk factors for obesity. P/B ratio was inversely related to fecal tryptophan, a metabolite related to serotonin biosynthesis, and positively related to nucleus accumbens centrality, a region central to the brain’s reward center. These findings may expand the field of therapies for obesity through novel pathways directed at the BGM axis.

## Introduction

Over the past 40 years, the global prevalence of obesity has increased dramatically to epidemic proportions, along with its associated health-related costs and risks, such as insulin resistance, type two diabetes, and cardiovascular disease.^1^ Studies aiming to determine the causes of obesity have demonstrated multifactorial contributors, including the influence of the environment, lifestyle and genetics, but especially dietary factors.^2^ The standard American diet, characterized by highly processed foods and dietary intake of saturated fats and low fiber, represents a growing health risk contributing to obesity.^3^ As diet is a reflection of one facet of socioeconomic determinants of health along with race, susceptibility to obesity is also influenced by race and ethnicity.^4^ In the United States, Hispanic adults (32.6%) experience the second highest prevalence of obesity next to non-Hispanic Blacks (38.4%), compared to non-Hispanic White adults (28.6%).^5^ While these disparities are influenced by socioeconomic disadvantages that intertwine with lifestyle and dietary factors, clinically and biologically modifiable risk factors involved in the development of obesity need to be investigated.

A plausible mechanism that can connect diet and life-style with obesity may be found within the brain-gut microbiome (BGM) axis, which is based on the concept that bidirectional signaling exists between the gut microbiome and the brain.^6^ Preclinical studies suggest that the microbiota is able to communicate with the brain through the release of neuroactive metabolites and thus manipulate host feeding behaviors (i.e. appetite, food preferences, etc.).^7^ For example, in a resting-state functional magnetic resonance imaging (fMRI) study with healthy participants, tryptophan-derived indoles produced by the microbiota were shown to influence hedonic food intake by acting on the network metrics of key regions within the brain’s reward network.^8^ Since the BGM axis is involved in regulating host metabolism, energy expenditure, and ingestive behaviors, perturbations anywhere along the axis can contribute to metabolic diseases, such as obesity.^6,7^ To our knowledge, there are very few if any obesity studies that apply a systems-biology approach to understand pathophysiological differences in the bidirectional BGM axis that may contextualize obesity while controlling for race and diet.

In order to accurately explore the effects of obesity on the BGM system, race, lifestyle, and dietary factors must be systematically accounted for, as these factors can influence the prevalence of obesity, even in groups with shared geography.^2^ Dietary intake varies with race along with corresponding differences in fecal bacteria and metabolites.^9^ In addition, diet creates interpersonal variations in microbiome composition, with a study reporting diet to influence 57% of total microbiome structural variations, compared to only 12% by genetic differences.^10–12^ Failing to disentangle the effects of diet, race, and obesity when studying the BGM axis prevents generalizable conclusions to be made on what aspects the BGM differences observed may contribute to increased risk for obesity.^13^

In order to characterize obesity-associated signatures in the BGM system, we explored interactions in the gut microbiome, metabolites, and brain network metrics using resting-state fMRI connectivity, while systematically adjusting for race and diet. We propose that a specific BGM signature exists independent of diet and race that is associated to obesity.

## Materials and methods

### Study participants

The study included 287 right-handed participants (99 males and 198 females), without any significant medical or psychiatric conditions. Participants were excluded for the following: pregnant or lactating, substance use, abdominal surgery, tobacco dependence (half a pack or more daily), extreme strenuous exercise (>8 h of continuous exercise per week), current or past psychiatric illness and major medical or neurological conditions, similar to our previous study.^14^ Participants taking medications that interfere with the central nervous system or regular use of analgesic drugs were excluded.^14^ Because of the effect of handedness on brain signatures and brain function related to laterality, only right-handed participants were included to exclude this as a cofounder.^15^ Included participants were also required to not have taken antibiotics for at least 3 months before enrolling in the study, similar to previously published works.^14^ Since female sex hormones, such as estrogen are known to affect brain structure and function, we used females who were premenopausal and who were scanned during the follicular phase of their menstrual cycles as determined by self-report of their last day of the cycle.^14^ Participants with hypertension, diabetes, metabolic syndrome or eating disorders were excluded to minimize confounding effects. We used body mass index (BMI) cutoffs to define our groups: Individuals with BMI < 30 were normal, and BMI ≥ 30 were considered obese. No participants exceeded 400 lbs due to magnetic resonance imaging (MRI) scanning weight limits. Participants underwent MRI scans, anthropometrics (height, body weight, and waist–hip ratio measurements, body mass index), and fresh stool samples for 16s ribosomal RNA gene sequencing and metabolite analysis were collected. Race and ethnicity were combined into a single category by making Hispanic a separate race category along with White, African American, Asian, and other.

### Diet questionnaires:

All participants underwent two surveys for their diet: Diet History Questionnaire (DHQ) III and the UCLA Diet Checklist. The DHQ-III is a validated food frequency questionnaire developed by the National Institute of Health to measure the frequency and portion size of foods consumed over a year.^16^ Diet*Calc software was used to analyze and interpret the raw DHQ-III data and determine nutrient and food group intake estimates. The Diet Checklist is a questionnaire developed by our institution and used in our previously published works.^14^ It is intended to represent the diet that best reflects what participants consume on a regular basis.^14^ The specific diets incorporated into this checklist are summarized in supplemental Table S1. This Diet Checklist was then internally validated against the standardized DHQ-III.^14^ For data analysis, we combined standard American and modified American diet as one category. Mediterranean, vegan, vegetarian, and gluten-free were each their own individual categories, and all other diets were combined as “other” for analysis, similar to our previous work.^14^

### Microbiome: 16S rRNA gene sequencing and analysis

Within 1 week of the participant’s brain MRI scan, stool samples were collected and stored at −80°C before 16S rRNA sequencing. DNA extraction was performed with the fecal samples using PowerSoil DNA Isolation Kit (MO BIO Laboratories, Carlsbad, CA) with bead beating following the manufacturers protocol. Using 515 F and 806 R primers, the V4 hypervariable region of the 16S rRNA gene were amplified. The PCR products were purified using the ZR-96 DNA Clean & Concentrator-5 Kit (Zymo Research, Irvine, CA) and subsequently sequenced with the Illumina HiSeq 2500 platform. Processing of base pair reads using QIIME v1.9.1 with default parameters and taxonomic sequences were assigned using closed reference operational taxonomic unit (OTU) picking in QIIME against the Greengenes database pre-clustered at 97% identity. If the OTUs were present in less than 10% of samples, they were removed. The median depth was 104,124 reads per sample with a standard deviation of 73,192 and a minimum read of 32,304.

### Enterotypes

Bacterial enterotypes were created based on the same methodology as previously published.^17^ The following packages were used in R: cluster, clusterSim, and ade4. Samples were clustered based on their relative abundances using a Jensen-Shannon distance metric and the Partitioning Around Medoids clustering algorithm. The optimal number of enterotypes was assessed using the Calinski-Harabasz index. The optimal number of enterotypes was validated using the Silhouette coefficients.^18^ A step-by-step tutorial can be found on the following site: https://enterotype.embl.de/enterotypes.html.

### Fecal metabolites

Using the same fecal samples as the 16S sequencing, samples were aliquoted under liquid nitrogen and then shipped to Metabolon. They were processed and analyzed as a single batch on Metabolon’s global metabolomics and bioinformatics platform. Using established protocols, data was curated by mass spectroscopy as previously reported.^19^ We specifically analyzed tryptophan-derived metabolites, due to their relevance in the BGM axis using the scaled imputed data from Metabolon.

### Brain MRI: acquisition

Whole brain structural and resting state functional connectivity data was collected using a 3.0 T Siemens Prisma MRI scanner (Siemens, Erlangen, Germany). Detailed information on the standardized acquisition protocols, quality control measures, and image preprocessing are provided in previously published studies.^14,20–25^

#### Structural MRI acquisition:

High resolution T1-weighted images were acquired: echo time/repetition time (TE/TR) = 3.26 ms/2200 ms, field of view^26^ = 220 × 220 mm, slice thickness = 1 mm, 176 slices, 256 × 256 voxel matrix, and voxel size = 0.86 × 0.86 × 1 mm.^14^

#### Resting-state functional MRI acquisition:

Whole brain resting state scans were acquired with eyes closed and an echo planar sequence with the following parameters: TE/TR = 28 ms/2000 ms, flip angle = 77°, scan duration = 10 m6s, FOV = 220 mm, slices = 40, and slice thickness = 4.0 mm.^14^

### Preprocessing of MRI images

Participants MRI images were preprocessed in a similar manner as to our previous work.^14^ In summary, preprocessing and quality control of functional images was done using SPM-12 software (Welcome Department of Cognitive Neurology, London, UK). The first two volumes were discarded to account for the approach toward steady-state magnetization.^25^ Slice timing correction was performed first, followed by rigid-body motion correction with six realignment parameters.^14^ If motion was detected above 2 mm translation or 2° rotation, the scan, along with the paired structural image, were discarded.^14^ To robustly account for the effects of motion, root mean squared (RMS)^27^ realignment estimates were calculated as robust measures of motion using publicly available MATLAB code from GitHub.^14,28^ Any participant with a RMS value greater than 0.25 was not included in the analysis.^14,28^ Each T1 image was then segmented and normalized to a template brain in Montreal Neurological Institute^27^ template space.^14^ Each participant’s T1 normalization parameters were then applied to that participant’s resting state images, resulting in an MNI space normalized resting state image.^14^ Complete detail is provided in previous published works.^14^

### Brain MRI: structural image parcellation

T1-image segmentation and cortical and subcortical regional parcellation were conducted using the Schaefer 400 atlas,^29^ the Harvard-Oxford subcortical atlas,^30,31^ and the Ascending Arousal Network atlas.^14,32^ This parcellation results in the labeling of 430 regions, 400 cortical structures, 14 bilateral subcortical structures, bilateral cerebellum, and 14 brainstem nuclei.^14,33^

### Brain MRI: resting-state functional brain connectivity matrix construction

Matrix construction was performed similarly to our previous work.^14^ Briefly, all pre-processed, normalized images were entered into the CONN-fMRI functional connectivity toolbox version 17^34^ and further corrected for noise using the automatic component-based noise correction (aCompCor) method to remove physiological noise without regressing out the global signal.^14,35^ Confounds for the six motion parameters with their first-order temporal derivatives, along with confounds emerging from white matter and cerebral spinal fluid, and root mean squared^27^ values of the detrended realignment estimates,^27^ were removed using regression.^14^ Connectivity matrices, containing all parcellated regions in the Schaefer,^29^ Harvard-Oxford Subcortical,^30,36–38^ and Ascending Arousal Network^27,32^ atlases, were then constructed. The final outputs for each participant consisted of a connectivity matrix between the 430 parcellated regions and was indexed by Fisher transformed Z correlation coefficients between each region of interest.^14^

### Brain MRI: computing resting-state network metrics

The Graph Theory toolbox (GTG) (www.nitrc.org/projects/metalab_gtg) and in-house MATLAB scripts were used to calculate and analyze the brain network properties and organization from the participant-specific resting-state functional brain networks for the brain. Network measures based on graph theory were investigated due to the emphasis on an integrated system versus on individual brain regions. Furthermore, we focused on measures of centrality, which are parameter-free methods that provide important insights about the level and quality of connectivity in specific regions. Mapping of both brain connectivity architecture allows for specificity and prediction of the functional roles of the brain regions. Brain regions with high centrality are highly influential, communicate with many other regions, facilitate functional integration, and play a key role in network resilience to insult.^39^ The three main indices of centrality were computed for the purposes of this project: 1) *Degree strength* (DS) reflects the number of other regions a brain region interacts with functionally (local prominence), *2) Betweenness Centrality* (BC) reflects the ability of a region to influence information flow (signaling) between two other regions, and 3) *Eigenvector Centrality* (EC), where higher values indicate the region is directly connected to other highly connection regions reflective of the global (vs. local) prominence of a region.

## Statistical analysis

The Kruskal–Wallis test was used for continuous variables and chi-squared test for categorical variables when analyzing baseline demographic characteristics differences. Medians were reported with their corresponding interquartile ranges. We calculated beta diversity using DEICODE plugin in QIIME 2, which accounts for sparse compositional nature of microbiome data with a robust Aitchison analysis. This method has been shown to yield higher discriminatory power compared to other common metrics, such as UniFrac or Bray-Curtis.^40^ Differences in beta diversity was calculated using the R package ‘adonis’ which implements a permutational analysis of variance. Alpha diversity was calculated in QIIME using OTU-level data rarefied to 32,303 sequences and significance was determined using Faith’s phylogenetic diversity (Faith’s PD), Chao1, and Shannon index by analysis of variance. Association of microbial genera were evaluated using DESeq2 in R, which uses an empirical Bayesian approach to minimize dispersion and fit non-rarified count data to a negative binomial model. Differential abundance p-values were converted to q-values to adjust for multiple hypothesis testing (<0.05 for significance). For metabolite and brain analyses (resting state functional connectivity measures of centrality for all 430 brain parcellations), data were first normalized and then analyzed using a generalized linear model (GLM) in R. Only results surviving FDR correction were reported.

Significant findings from fMRI, metabolite, 16S microbiome, and clinical data were combined into one dataset, and spearman correlations between datapoints were performed using the *Hmisc* and *corrplot* packages in R. All p-values were adjusted for multiple hypothesis testing using false discovery rate correction. Correlation networks were then visualized using Circos plots.^41^

## Results

### Population demographics

The study cohort included 287 participants and consisted of obese (n = 81; median BMI = 33.6 [IQR 31.9–36.6]) and non-obese individuals (n = 216; median BMI = 23.9 [IQR 21.5–26.2]) (p <.001). The median age of the obese cohort was significantly older, which was 32 years for the obese group and 26 years for the non-obese group (p <.001; Table 1). The majority of the participants were female (n = 198) and non-Hispanic White (n = 110), followed by Hispanic (n = 92), Asian (n = 65), Black (n = 24), and Native American(n = 6). There were significant differences in race by obesity (p < .001), with Hispanics (53.1%) representing the majority in the obese and non-Hispanic Whites (41.7%) as the largest group in the non-obese cohort. There were significant differences based on diet in the obese and non-obese groups (p = .037), with more individuals on a standard American diet (82.7%) in the obese group as compared to the non-obese group (59.7%). Three microbiome enterotypes were identified. These three enterotypes were based on their differences in their relative abundances of *Prevotella* and *Bacteroides*. Therefore, a *Prevotella/Bacteroides* (P/B) ratio was created as an index for the enterotype clusters. Participants with the highest tertile with regard to the P/B ratio were more likely to be obese (p = .002).

**Table 1.** Population characteristics

	Normal/Overweight (n = 216)	Obese (n = 81)	p-value
Age (yr) (median, IQR)	26 [21–34]	32 [26–41.5]	<0.001
Gender (n, %)			
Female (n = 198)	139 (64.4%)	59 (72.8%)	0.17
Male (n = 99)	77 (35.6%)	22 (27.2%)	
Race (n, %)			
Asian (n = 65)	60 (27.8%)	5 (6.2%)	<0.001
Black (n = 24)	11 (5.1%)	13 (16.1%)	
Hispanic (n = 92)	49 (22.7%)	43 (53.1%)	
Indian (n = 6)	6 (2.8%)	0 (0.0%)	
Non-Hispanic White (n = 110)	90 (41.7%)	20 (24.7%)	
BMI (median, IQR)	23.9 [21.5–26.2]	33.6 [31.9–36.6]	<0.001
Diet Category (n, %)			
Standard American (n = 196)	129 (59.7%)	67 (82.7%)	0.037
Vegetarian (n = 30)	25 (11.6%)	5 (6.2%)	
Vegan (n = 4)	3 (1.4%)	1 (1.2%)	
Mediterranean (n = 23)	20 (9.3%)	3 (3.7%)	
Gluten Free (n = 6)	4 (1.9%)	2 (2.5%)	
Other (n = 38)	35 (16.2%)	3 (3.7%)	
*Prevotella*/*Bacteroides* Ratio Tertiles (n, %)			
Low (n = 98)	84 (38.9%)	14 (17.3%)	0.002
Mid (n = 98)	66 (30.6%)	32 (39.5%)	
High (n = 101)	66 (30.6%)	35 (43.2%)	

Participant characteristics by obesity status. Continuous variables are presented as median with their respective interquartile range (IQR). Percentages listed are percent of the total number of normal/overweight or obese participants. BMI: Body mass index. Bolded p-values are significant p-values (p-values<0.05). Significance of categorical data was determined by chi-squared test and significance of continuous variables was determined by Kruskal–Wallis test.

### Microbial differences based on race

In order to identify the independent effects of race on microbial diversity, samples were stratified by race and analyses were performed using the 16S rRNA sequence data while adjusting for covariates, such as obesity, diet, and sex. Overall, microbial composition, as determined by beta-diversity, showed highly significant differences across samples stratified by race (p < .001; Fig. S1A). Post-hoc pairwise testing showed that there were beta diversity differences between Asians and Blacks (p = .013), Asians and Hispanics (p = .017), and Asian and NHW (p = .013). There were no significant differences between Black and Indians (p = .073), Black and NHW (p = .073), and Hispanics and NHW (p = .073) (Supplemental Table S2). When comparing species evenness as measured by the abundance of organisms across species (Shannon index), there were also differences across race (p = .004) (Fig. S1B). Post-hoc pairwise testing showed that NHW had higher Shannon index as compared to Asians (p = .004). There were no other significant pair-wise difference in regard to the Shannon index. (Supplemental Table S2).

### Microbial differences based on diet

Because the majority of participants were on a standard American diet, we dichotomized the group into two categories: those who were on a standard American diet versus those who were not. Observed differences in diet between the obese and non-obese cohorts prompted us to compare microbiome differences based on standard American diet, while adjusting for obesity, race, and sex. There were observable taxonomic differences in the relative abundance of genera based on diet (standard American diet vs. nonstandard American diet) and several taxa significantly increased or decreased in association with standard American diet (Fig. S2A, B). Differential abundance testing demonstrated that 12 genera differed between individuals based on diet (q-values < 0.05) (Fig. S2B). After adjusting for race and obesity effects, microbial composition was associated with standard American diet (p =.048; Fig. S2C). However, no statistical differences in Shannon index based on diet were observed (p =.14) (Fig. S2D).

### Microbiome differences based on obesity, adjusting for race, diet, and sex

**Figure 1.**
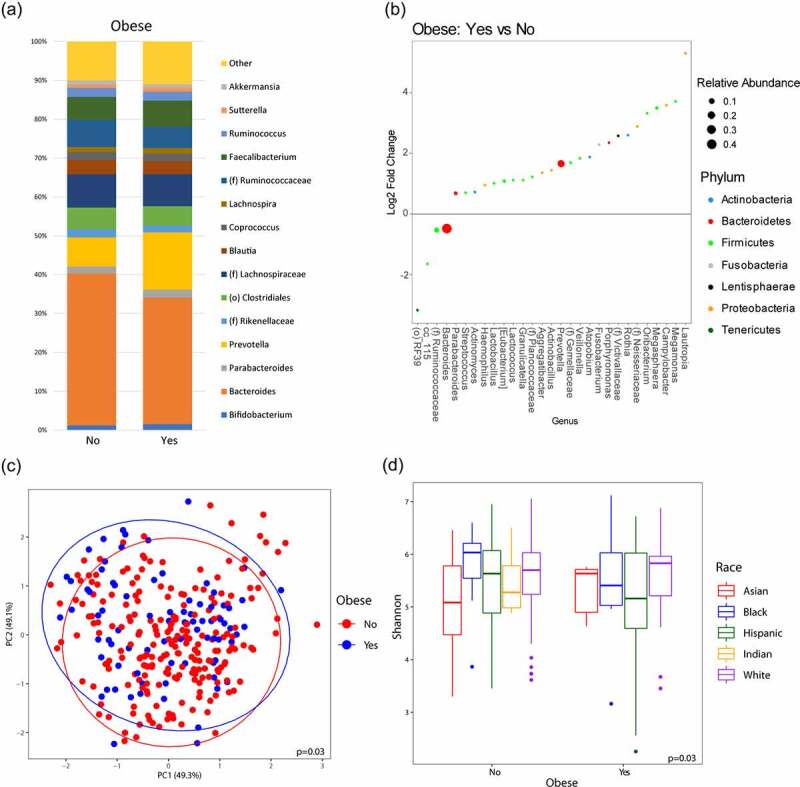
**Microbiome diversity and composition varies with obesity**. (a) Taxonomic summary plots showing relative abundance of all genera (minimum of 1% relative abundance) grouped by obesity, adjusting for sex, race and diet. (b) Log2 fold changes for genera with differential abundance between obese vs. non-obese in DESeq2 models (q < 0.05), adjusting for race and diet. (c) Principal coordinate analysis plot of the microbiome based on obesity encircled by 99% confidence interval ellipses, adjusting for sex, race and diet. (d) Box plot of microbial diversity by Shannon index (measure of richness and evenness) grouped by obesity and stratified by race, adjusting for race, diet, and sex.

When adjusting for the effects of race, diet, and sex on the microbiome, there were observed differences in genera associated solely with the effects of obesity, which was defined as a BMI ≥ 30. Of note, increased *Prevotella* (q-value = 0.004) and decreased *Bacteroides* (q-value = 0.03) were associated with obesity (Figure 1a, b). After adjusting for covariates, there were significant differences in overall microbial composition with obesity (p =.03; Figure 1c). When examining alpha diversity comparisons using the Shannon index across obesity, race-specific differences were observed (p =.03; Figure 1d). Post-hoc pairwise testing showed that Asian without obesity had lower Shannon index than NHW without obesity (p =.03). No other pairwise comparison were significant after adjusting for false discovery rate. There was also no significant interaction between race and obesity on Shannon index (p = .19).

### Enterotype-based differences in microbiome diversity and composition that correlates with BMI

Three distinct microbial profiles were observed in the samples that were driven by either a relatively high abundance of the genera *Prevotella* (Enterotype A), *Bacteroides* (Enterotype C), or a mixed population (Enterotype B) after adjusting for covariates, such as race, diet, obesity, and sex (Fig. S3A). The number of differentially abundant genera for each comparison were as follows: 21 genera between enterotypes A and B, 55 genera between enterotype A and C, and 50 genera between enterotype B and C (q-values < 0.05) (Fig. S3B-D). The relative abundance of *Prevotella* correlated positively with enterotype A and B compared to enterotype C (q-value < 0.001). However, increases in *Bacteroides* correlated negatively with enterotype A and B, compared to enterotype C (q-value < 0.001). When comparing enterotype A and B, *Prevotella* (q-value < 0.001) was more highly enriched and *Bacteroides* (q-value < 0.001) was less abundant in enterotype A. Adjusting for covariates, such as race, diet, obesity, and sex, the microbial profile differed significantly between the three observed enterotypes (p < .001; Fig. S3E). Post-hoc pairwise testing showed that all enterotypes were different from each other (A vs B (p <.001), A vs C (p <.001), B vs C (p <.001)). Differences in Shannon index between the three enterotypes were also observed, with enterotype B demonstrating the highest alpha diversity (p <.001; Fig. S3F). Post-hoc pairwise testing showed that all enterotypes also were different from each other by Shannon index (A vs B (p < .001), A vs C (p = .01), B vs C (p <.001). As mentioned previously, because the enterotypes were based on differences between *Prevotella* and *Bacteroides*, a P/B ratio was created as an index measurement for the enterotype clusters.

**Figure 2.**
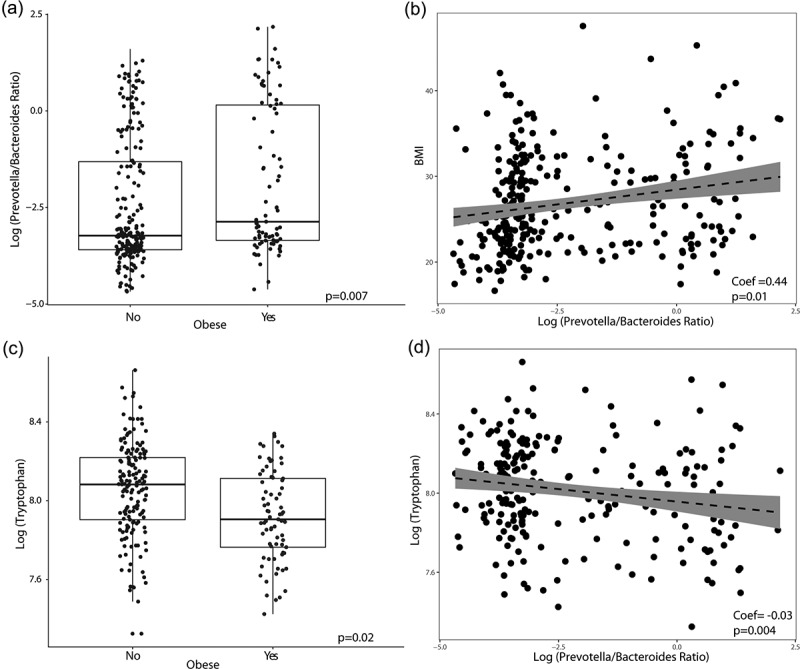
**Obesity is associated with elevated *Prevotella*/*Bacteroides* (P/B) ratio and lower fecal tryptophan**. (a) Boxplot of *Prevotella*/*Bacteroides* ratio grouped by obese and non-obese. (b) Linear regression between BMI and P/B ratio. (c) Boxplot of fecal tryptophan levels grouped by obese and non-obese. (d) Linear regression between tryptophan and P/B ratio. All p-value listed are adjusted for race, diet, and sex.

Upon examining the relationship between P/B ratio and obesity, P/B ratio was significantly different between the obese and non-obese groups (p =.007) (Figure 2). In a linear regression model adjusting for sex, race, and diet, P/B ratio was positively correlated to BMI (coef = 0.44, p =.01).

### Fecal tryptophan levels differ with obesity

After adjusting for sex, race, standard American diet, and false discovery rate, fecal tryptophan levels were significantly lower with obesity (p =.02). In a linear regression model adjusting for sex, race, and diet, fecal tryptophan was also negatively correlated with P/B ratio (coef = −0.03, p =.004) (Figure 2d).

### Brain region network metric measures differ with obesity

**Figure 3.**
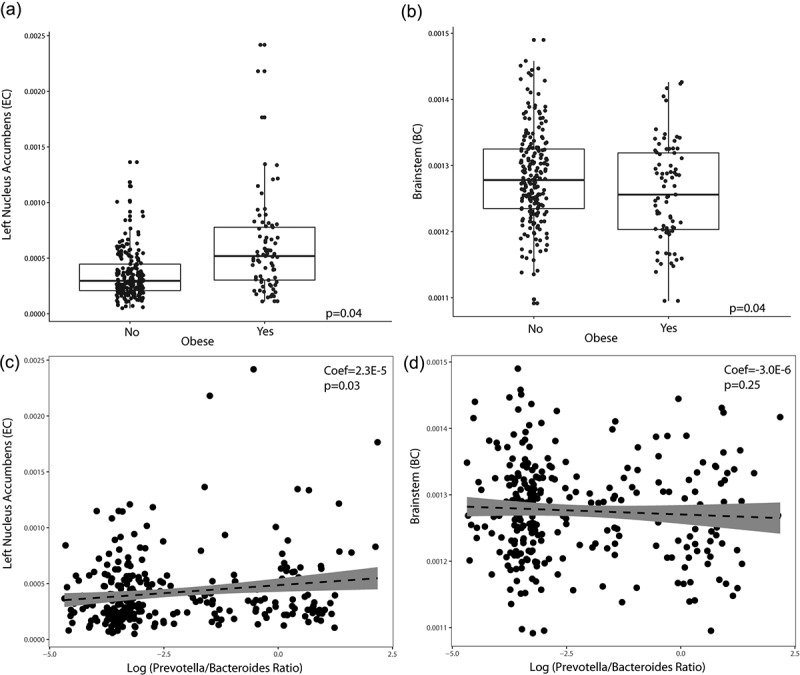
**Changes in brain measures by obesity and *Prevotella*/*Bacteroides* (P/B) ratio**. (a) Brain network metric (Eigenvector Centrality; EC) levels of the left nucleus accumbens by obesity. P-value listed is adjusted for sex, race and diet and false discover rate. (b) Brain network metric (Betweenness Centrality; BC) levels of the brainstem by obesity. P-value listed is adjusted for sex, race and diet and false discover rate. (c) Linear regression between the left nucleus accumbens and P/B ratio adjusting for sex, race and diet. (d) Linear regression between the brainstem and P/B ratio adjusting for race, diet, and sex.

After adjusting for the covariates sex, race, diet, and false discovery rate, obesity was positively associated with left nucleus accumbens (p =.04) eigenvector centrality, and negatively associated with brainstem (p =.04) betweenness centrality (Figure 3). No other centrality measurements for the nucleus accumbens or brainstem were significantly associated with obesity (p >.05). A positive correlation was also seen between P/B ratio and left nucleus accumbens eigenvector centrality from our linear regression model adjusting for sex, race, and diet (coef =2.3E-5, p =.03) (Figure 3c). There was no linear relationship between brainstem betweenness centrality and P/B ratio (p =.25). We then evaluated if there was an interaction effect between race and obesity on P/B ratio and nucleus accumbens eigenvector centrality adjusting for sex and diet using GLM (i.e., ~ sex + diet + race*obesity). Because NHW and Hispanics made the vast majority of the sample population, we focused this model to only those races in order to minimize over-parameterization. In this model, there was no significant interaction effect of race and obesity on P/B ratio (p =.98) or nucleus accumbens centrality (p =.17) after adjusting for sex and diet.

### P/B ratio and nucleus accumbens centrality are independent risk factors for obesity

**Figure 4.**
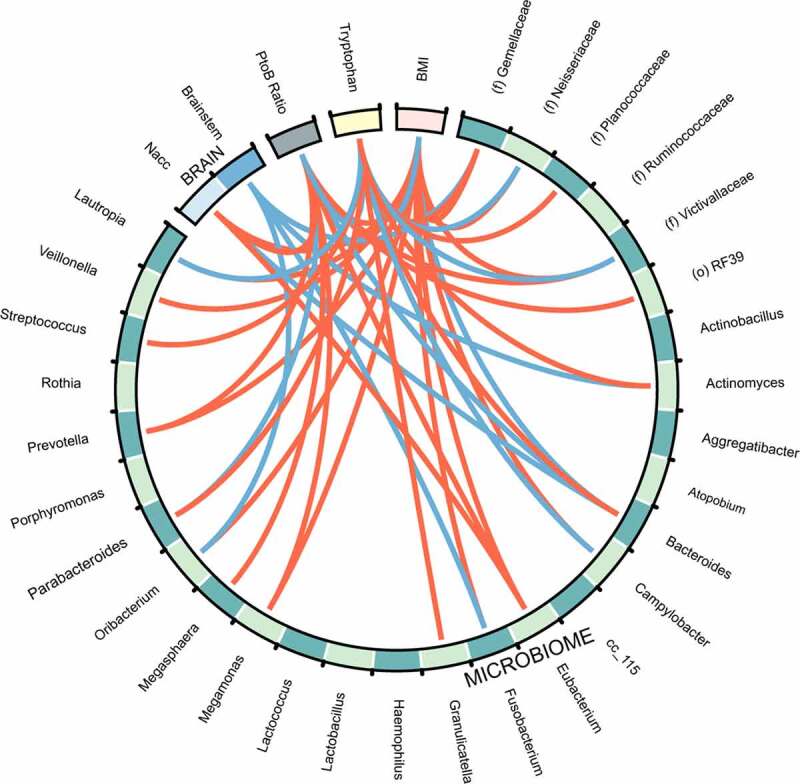
**Circos plot of all datasets**. Red lines indicate positive correlations and blue lines indicate negative correlations (False discovery rate cutoff = 0.10). Bacteria to Bacteria associations were excluded from this plot for easier viewing. Nacc: Nucleus accumbens; BMI: Body mass index. P/B: *Prevotella*/*Bacteroides*. The strength of the correlations can be found in supplemental table S3.

We then performed logistic regression to examine the effects of sex, race, diet, P/B ratio, and nucleus accumbens centrality on obesity. Nucleus accumbens activity and P/B ratio were split into tertiles. Sex did not affect the risk of having obesity (p =.33). However, being Hispanic (p <.001), eating a standard American diet (p =.003), having a high P/B ratio (p =.02) and high nucleus accumbens centrality (p =.002) were all independent risk factors for obesity. There was no interaction effect between high nucleus accumbens and high P/B ratio on obesity (p =.22) (Table 2). Spearman correlations of the significant brain regions, clinical data, and 16S sequencing are summarized in the Circos plot seen in Figure 4. From the Circos plot, after adjusting for multiple hypothesis testing, we see that, overall, there are significant interactions between BMI and the BGM axis. BMI had significant interactions with P/B ratio, nucleus accumbens centrality, fecal tryptophan, and several genera. Furthermore, P/B ratio, nucleus accumbens centrality, and fecal tryptophan all had significant interactions with each other.

**Table 2.** Odds of having obesity by *Prevotella*/*Bacteroides* ratio and nucleus accumbens centrality

Variable	OR	95% CI	p-value
Male	0.69	−0.06–1.44	0.33
Standard American Diet	3.35	2.55–4.14	0.003
Hispanic	4.67	3.97–5.38	<0.001
High P/B Ratio	2.64	1.83–3.46	0.02
High Nacc	3.52	2.74–4.31	0.002
High P/B Ratio*High Nacc	0.23	−2.16–2.62	0.22

Output of a logistic regression model for obesity. Eigenvector centrality (EC)of the left nucleus accumbens and the log(*Prevotella*/*Bacteroides*) ratio were separated into tertiles and the presence of the highest tertile was used in the model (High Nacc and High P/B Ratio, respectively). The model was adjusted for other covariates (i.e., obesity ~ sex + diet + race + High P/B ratio + High Nacc + High P/B ratio*High Nacc). The odds ratio (OR) for having obesity is reported along with their respective 95% confidence interval (CI). Significant p values are bolded (p < 0.05).

## Discussion

Although there has been an increasing focus on exploring the disease processes underlying obesity by examining the gut microbiome and brain, there are few studies that utilize an integrative approach to understand the interactions within the BGM axis in obesity, and even fewer that do so while adjusting for known confounding factors that impact the BGM axis. In this study, we demonstrated that race and diet have independent effects on microbiome diversity regardless of obesity state, and subsequently characterized the BGM axis changes associated with obesity while adjusting for these demonstrated covariates. These finding are of great interest given the increasing efforts to understand the pathophysiology of obesity for the development of novel therapies and regimens.

Previous initiatives to characterize the microbiome in various ethnic and geographical populations have reported pronounced differences in microbial communities.^42^ However, many of these studies compare groups across geographical borders, which introduces broad differences in environmental and lifestyle factors that confound the findings. The findings from the few studies that compare ethnicity within a country support results from our study. In a Canadian study that compared the gut microbiomes of Caucasian and South Asian infants, differences were observed in alpha diversity (Shannon index) and beta diversity (Bray-Curtis dissimilarities) between the two races, which is consistent with our findings of alpha and beta diversity differences based on race.^43^ We found that Asians have the lowest alpha diversity by Shannon index and that it is significantly lower than Whites (p <.004). This is in agreement with the findings of an United States-based study, which also reported that Asian-Pacific Islanders had significantly lower Shannon diversity compared to Caucasians and Hispanics.^44^

In prior research, long-term dietary habits influence gut microbiota composition, notably when comparing fiber versus protein-based diets. Our study showed that there were several taxonomic changes associated with a standard American diet, which is a low-fiber diet that is high in refined carbohydrates, fatty meats, and saturated fats. Of note, individuals on standard American diet were found to have decreased *Lachnospira*, which has been positively associated with vegetarian diets and negatively associated with higher meat and cholesterol intake.^45^ *Ruminococcus* has also been shown to have correlations with a protein-rich diet, and we similarly saw increases in this genus with those individuals on the standard American diet.^45^ In addition, we observed that decreased *Bacteroides* and increased *Prevotella* abundance were also associated with a standard American diet. *Prevotella*-rich microbiomes are associated with insulin resistance through the production of branched chained amino acids, especially in those on a high-fat diet, such as a standard American diet, and potentially contributes to the increased risk of developing insulin resistance in obese individuals.^46^

Although there have been many preclinical and clinical studies that have aimed to characterize the obese microbiome, these results have shown inconsistencies. While initial studies in mice demonstrated that the microbiota of obese mice is enriched in Firmicutes and reduced in Bacteroidetes relative to that of lean mice, studies in humans have demonstrated similar findings,^47^ no associations,^48^ or contradictory results.^49^ Later, meta-analyses demonstrated that these associations were either very small or non-existent.^50,51^ In addition to comparing broad taxonomic groups, instead of genera or species, these previous studies failed to account for interpersonal variations that exist in the microbiome due to confounding factors, with the most prominent factors being diet and race.

In our study, we were able to account for these covariates and observed significant taxonomic changes at the genus level associated with obesity, including enrichments in *Prevotella* and decreases in *Bacteroides*, which is also supported by previous studies.^52^ Interestingly, while *Prevotella* and *Bacteroides* are closely related functionally and are able to individually dominate the gut microbiome, they are rarely found together in high relative abundance and seem to exhibit a co-exclusionary relationship.^53^ In vitro studies have shown that *Prevotella copri*, which belongs to the genus *Prevotella*, is able to stimulate pro-inflammatory cytokines such as IL-6, IL-23, and IL-17, and perpetuate an inflammatory environment.^54^ Mice that are colonized with *P. copri* also experience exacerbated colitis and overall more epithelial damage due to inflammation.^55^ Obesity is considered a chronic low-grade inflammatory disease with elevated circulating pro-inflammatory cytokines, which may be maintained by a pro-inflammatory *Prevotella*-driven microbiome.^56^ This inflammatory state in obesity is also seen in relation to microbial gene richness, as individuals with lower diversity tend to carry genes associated with oxidative stress.^57^

Our data also shows an inverse relationship between fecal tryptophan and obesity, a finding that is consistent in prior research.^58^ Tryptophan is directly related to the biosynthesis of important neurotransmitters, such as serotonin and melatonin and several studies, have shown a negative correlation between tryptophan levels and obesity.^58,59^ The proposed mechanism by which this occurs is the shunting of dietary tryptophan toward pro-inflammatory kynurenine metabolites, adding to the proinflammatory state of obesity.^58,59^ The changes seen in a metabolite that is very directly related to brain function and signaling, like tryptophan, emphasize the central nature that the BGM axis plays in obesity.

This is particularly evident when we examine the findings related to the brain. Our results show an increased relationship of eigenvector centrality of the nucleus accumbens to both obesity and P/B ratio. In children with a genetic polymorphism for obesity, larger nucleus accumbens volumes were detected along with responsivity to food cues compared to lower-obesity risk controls.^60^ The nucleus accumbens is involved in regulating the reward aspects of food intake and dysregulation can lead to addiction-like behaviors resulting in weight gain.^61^ Increased activity in reward-regions in the brain are also predictive of weight gain in adulthood.^62^ Eigenvector centrality reflects how a region is directly connected to other regions of high connectivity, which is reflective of its global importance in other networks. Because DS and BC of the nucleus accumbens was not associated with obesity, this suggests that the nucleus accumbens role in obesity is more reflective of its interactions globally throughout the brain instead of its local interaction or ability to influence local signaling. In addition to finding increased measures of centrality in the nucleus accumbens in our obese group, we also observed decreased measures of betweenness centrality in the brainstem. Betweenness centrality is measure that reflects the ability of a region to affect the signal of another region. This is not surprising as the brainstem is recognized to be involved in food intake modulation by receiving inputs from the gut through cholecystokinin and then sending dopaminergic projections to the hypothalamus.^63^ Lower brainstem betweenness centrality suggests disruptions in this homeostatic network conveying these signals of satiety. A study comparing subcortical volumes involved in food intake similarly found decreased brainstem volumes in the obese group relative to controls.^64^ With DS and EC of the brainstem not being related to obesity suggests that the way the brainstem influence local signaling is more important to obesity and not the number of regions it is locally or globally connected to. There has been some discussion about the correlation between measures of centrality, and the assumption is that since these metrics are derived from the same data matrix they should be redundant or highly related. For example, some studies have demonstrated that degree centrality is correlated with eigenvector centrality but not betweenness centrality. However, studies have also shown that these measures do have distinct functional properties and measure different types of information flow and connectivity that are associated with specific outcomes, circumstances, and diseases. Hence, data should be interpreted as such (regardless of the possible underlying similarities in the underlying data).^65–68^ With larger samples other measures of brain network metrics, such as segregation and integration, and their relationships to each other could be investigated, which would further highlight functional properties of connectivity in these key regions. Since a whole brain approach may limit detection of significant results, future studies can also take a region of interest approach and use these measures in collaboration with other MRI modalities to provide more comprehensive inferences and insights.

While we demonstrated that diets such as the standard American diet may contribute to a microbiome state with increased susceptibility to obesity, the association of the microbiome to an elevated BMI were also independent of diet. From our analysis, we see that the prevalence of an elevated P/B ratio and a higher level of nucleus accumbens centrality was independently associated with obesity. In our GLM model, the lack of an interaction effect of race and obesity on either P/B ratio or nucleus accumbens centrality suggests that the effects of having either of these risk factors increases one’s risk for obesity irrespective of race. This is evident in our logistic regression, which shows race, diet, high P/B ratio, and high nucleus accumbens centrality as independent risk factors for obesity. However, being Hispanic was still an independent risk factor for obesity irrespective of P/B ratio or nucleus accumbens centrality. This suggests that other variables relating to race outside of the BGM axis still play a significant role in the development of obesity, warranting further research.

While our study has several strengths including its relatively large size of 287 participants with a comprehensive dataset and consideration of major covariates, there are also several limitations, such as the cross-sectional nature of our study design. The self-identification method used for collecting racial data may also not adequately account for the multidimensional construct of race, which may contribute to within-group heterogeneity.^69^ Future studies incorporating metagenomic methods to detect human genetic variants seen with race may help account for this heterogeneity.^44^ The utilization of 16S rRNA sequencing also inherently has its disadvantages due to its low resolution, which limits data interpretation in terms of species and strain level functional analyses.^70^ In future controlled studies, additional covariates, such as early-life experiences and early childhood exposure to antibiotics may also need to be accounted for, as these are both factors with demonstrated influence on the microbiome.^71^

Despite the growing health burden of obesity worldwide, drug development efforts and proposed therapeutic strategies for obesity have yielded disappointing results, with many only producing modest reductions in weight and frequent refractory outcomes.^72,73^ While the health disparities related to obesity in the United States are frequently attributed to dietary factors, our data suggest there may be other pathophysiological factors in the brain and gut microbiome that affect obesity, paving the way for personalized and earlier interventions in these individuals with identified obesity risk signatures in their BGM axis. In addition, our study proposes the utility of a multimodal approach for targeting obesity, which includes modalities that target the brain (i.e. cognitive-behavioral therapy, dietary counseling, deep brain stimulation), gut microbiome alterations (i.e. bariatric treatments, fecal microbiota transplantations, pre/post-biotics), and the BGM axis (i.e. postbiotics using metabolites such as tryptophan-derived compounds) itself.^7,74–76^ Additional controlled studies using a multi-omics approach and advanced brain imaging techniques are warranted to determine the causal roles of the gut microbiome and specific brain regions in obesity.

## Conclusion

In conclusion, we show that there is an obesity-related signatures in the BGM-axis independent of sex, race, and diet. Race, diet, P/B ratio and increased nucleus accumbens were independent risk factors for obesity. P/B ratio was inversely related to fecal tryptophan, a metabolite related to serotonin biosynthesis, and positively related to nucleus accumbens centrality, a region central to the brain’s reward center. Potential pharmacological targets of the BGM-axis such as neuromodulators or probiotics may be new avenues to treat obesity.

## Supplementary Material

Supplemental Material

## Data Availability

The datasets generated during and/or analyzed during the current study are not publicly available due to an ongoing collaboration with multiple principal investigators involving participant identifiers at the G. Oppenheimer Center for Neurobiology of Stress and Resilience. But data are available from the corresponding author on reasonable request.

## List of Abbreviations

Automatic component-based noise correction (aCompCor)

Betweenness Centrality (BC)

Body mass index (BMI)

Brain-gut microbiome (BGM) axis

Cerebral spinal fluid (CSF)

Degree strength (DS)

Diet History Questionnaire (DHQ)

Eigenvector Centrality (EC)

Graph Theory GLM toolbox (GTG)

Non-Hispanic Whites (NHW)

Operational taxonomic unit (OTU)

Root mean squared (RMS)
