# Supplementary material for: Obesity is associated with a distinct brain-gut microbiome signature that connects Prevotella and Bacteroides to the brain’s reward center
Source: Gut Microbes. 2022 Mar 20;14(1):2051999. doi: 10.1080/19490976.2022.2051999 (PMC8942409; doi:10.1080/19490976.2022.2051999)
Supplement: Supplemental Material [file KGMI_A_2051999_SM9661.docx]

**Supplementary Materials**

***Supplementary Tables***

**Table S1. Diet Checklist Categories**

| **Diet Category** | **Components** |
| --- | --- |
| Standard American | High consumption of processed foods, pastas, and breads. Meats, including red meat, fish, eggs, and dairy products consumed.  Vegetables and fruits consumed, but not in large quantities |
| Modified American | High consumption of processed foods, pastas, and breads (mainly whole grain). Poultry, fish, eggs, and dairy products consumed. Red meat consumed in limited quantities  Vegetables and fruits consumed, but not in large quantities |
| Mediterranean | High consumption of fruits, vegetables, bread and other cereals, beans, nuts, and seeds.  Olive oil is the key monounsaturated fat source.  Dairy products, fish, and poultry are consumed in low to moderate amounts. Little red meat is consumed. Eggs are eaten zero to four times a week and wine is drunk in moderate (or low) amounts. |
| Paleo | Consumption of basic foods such as plain meat, fish, shellfish, eggs, nuts, vegetables, fruits, berries, and mushrooms.  Minimally processed oils, such as avocado, olive or coconut oi, for cooking.  Excludes dairy products, legumes, dry beans, grains, coffee, alcohol, sugar, and processed foods. |
| Vegan | Focus is on plant-based foods. Includes fruits, vegetables, dried beans and peas, grains, seeds, and nuts. Excludes all meat and animal products. |
| Vegetarian  (6 categories) | Focus is on plant-based foods. Includes fruits, vegetables, dried beans and peas, grains, seeds, and nuts. |
| Vegetarian | Excludes all meat but will allow animal-derived ingredients, ie. honey and gelatin |
| Lacto-Vegetarian | Includes plant foods plus dairy products, no eggs |
| Ovo-Vegetarian | Includes plant foods plus eggs, no dairy |
| Lacto-Ovo-Vegetarian | Includes both dairy products and eggs |
| Pescatarian | Includes fruits, vegetables, dried beans and peas, grains, seeds, and nuts. Excludes all meat except fish. |
| Raw Vegan/ Raw Food | Unprocessed vegan foods that have not been heated above 115 degrees Fahrenheit (46 degrees Celsius) |
| Gluten-Free | Diet includes most foods but avoids the protein gluten, which is found in wheat, barley, and rye. |
| Dairy-Free | Diet includes most foods but avoids dairy. |
| Low FODMAP (Fermentable Oligo-,Di-, Monosaccharides, and Polyols) | Diet limits foods high in sugar and carbohydrates (fructose, lactose, fructans, galactans, and polyols). |

| Beta Diversity Post Hoc Pairwise Testing | | | Alpha Diversity Post Hoc Pairwise Testing | | |
| --- | --- | --- | --- | --- | --- |
| Variable | Variable | FDR p-value | Variable | Variable | FDR p-value |
| Asian | Black | 0.013 | Asian | Black | 0.06 |
| Asian | Hispanic | 0.017 | Asian | Hispanic | 0.36 |
| Asian | Indian | 0.873 | Asian | Indian | 0.86 |
| Asian | White | 0.013 | Asian | White | 0.004 |
| Black | Hispanic | 0.273 | Black | Hispanic | 0.6 |
| Black | Indian | 0.073 | Black | Indian | 0.99 |
| Black | White | 0.073 | Black | White | 0.99 |
| Hispanic | Indian | 0.286 | Hispanic | Indian | 0.99 |
| Hispanic | White | 0.073 | Hispanic | White | 0.36 |
| Indian | White | 0.3 | Indian | White | 0.99 |

**Table S2:** Post hoc pair-wise analysis of beta diversity and alpha diversity of Fig S1. FDR: False Discovery Rate.

| row | column | cor | p | FDR |
| --- | --- | --- | --- | --- |
| Streptococcus | Granulicatella | 0.51531 | 0 | 0 |
| Actinomyces | Granulicatella | 0.551765 | 0 | 0 |
| Granulicatella | (f) Planococcaceae | 0.477959 | 0 | 0 |
| Actinomyces | Rothia | 0.521822 | 0 | 0 |
| Haemophilus | Veillonella | 0.594593 | 0 | 0 |
| Actinobacillus | Veillonella | 0.469424 | 0 | 0 |
| Haemophilus | Actinobacillus | 0.814579 | 0 | 0 |
| Aggregatibacter | Actinobacillus | 0.700533 | 0 | 0 |
| Streptococcus | Actinomyces | 0.571567 | 0 | 0 |
| Haemophilus | Aggregatibacter | 0.703122 | 0 | 0 |
| P/B ratio | Bacteroides | -0.63728 | 0 | 0 |
| P/B ratio | Prevotella | 0.898331 | 0 | 0 |
| Bacteroides | Parabacteroides | 0.45662 | 6.66E-16 | 3.05E-14 |
| Granulicatella | (f) Gemellaceae | 0.450016 | 1.78E-15 | 7.55E-14 |
| Actinomyces | (f) Gemellaceae | 0.447913 | 2.66E-15 | 1.06E-13 |
| Actinomyces | (f) Planococcaceae | 0.440372 | 8.44E-15 | 3.14E-13 |
| Granulicatella | Rothia | 0.437449 | 1.31E-14 | 4.33E-13 |
| Aggregatibacter | Veillonella | 0.437846 | 1.24E-14 | 4.33E-13 |
| Streptococcus | (f) Gemellaceae | 0.431994 | 3.00E-14 | 9.39E-13 |
| Streptococcus | Rothia | 0.403368 | 1.85E-12 | 5.51E-11 |
| Streptococcus | Veillonella | 0.400927 | 2.59E-12 | 7.33E-11 |
| Streptococcus | (f) Planococcaceae | 0.397109 | 4.33E-12 | 1.17E-10 |
| (f) Planococcaceae | (f) Gemellaceae | 0.38885 | 1.30E-11 | 3.35E-10 |
| Veillonella | Campylobacter | 0.387576 | 1.53E-11 | 3.79E-10 |
| Streptococcus | Lactococcus | 0.382288 | 3.03E-11 | 7.20E-10 |
| Actinomyces | Veillonella | 0.369304 | 1.54E-10 | 3.51E-09 |
| Actinomyces | Lactococcus | 0.36427 | 2.83E-10 | 6.23E-09 |
| (o) RF39 | (f) Ruminococcaceae | 0.360777 | 4.29E-10 | 9.12E-09 |
| Haemophilus | Rothia | 0.344932 | 2.68E-09 | 5.49E-08 |
| (f) Planococcaceae | Atopobium | 0.337797 | 5.91E-09 | 1.17E-07 |
| Bacteroides | Prevotella | -0.33724 | 6.28E-09 | 1.21E-07 |
| (f) Gemellaceae | Rothia | 0.33373 | 9.20E-09 | 1.71E-07 |
| (f) Ruminococcaceae | Lactobacillus | 0.332451 | 1.06E-08 | 1.90E-07 |
| (f) Planococcaceae | Rothia | 0.327737 | 1.74E-08 | 3.05E-07 |
| Veillonella | Oribacterium | 0.325065 | 2.31E-08 | 3.93E-07 |
| Aggregatibacter | Campylobacter | 0.323624 | 2.69E-08 | 4.44E-07 |
| Streptococcus | Lactobacillus | 0.320991 | 3.53E-08 | 5.67E-07 |
| Actinobacillus | Rothia | 0.319647 | 4.05E-08 | 6.34E-07 |
| Tryptophan | (f) Victivallaceae | -0.36321 | 4.18E-08 | 6.38E-07 |
| Veillonella | Rothia | 0.307457 | 1.38E-07 | 2.05E-06 |
| Haemophilus | Campylobacter | 0.304399 | 1.86E-07 | 2.69E-06 |
| Veillonella | Fusobacterium | 0.303405 | 2.04E-07 | 2.89E-06 |
| Lactococcus | (f) Gemellaceae | 0.297939 | 3.45E-07 | 4.77E-06 |
| P/B ratio | (f) Victivallaceae | 0.289774 | 7.38E-07 | 9.98E-06 |
| (o) RF39 | (f) Victivallaceae | 0.288621 | 8.20E-07 | 1.08E-05 |
| Actinomyces | Oribacterium | 0.288161 | 8.55E-07 | 1.11E-05 |
| Actinobacillus | (f) Gemellaceae | 0.286774 | 9.70E-07 | 1.23E-05 |
| Campylobacter | Lautropia | 0.286386 | 1.00E-06 | 1.25E-05 |
| Haemophilus | (f) Gemellaceae | 0.279237 | 1.90E-06 | 2.31E-05 |
| Actinomyces | Eubacterium | 0.277359 | 2.24E-06 | 2.67E-05 |
| Granulicatella | Atopobium | 0.275291 | 2.69E-06 | 3.13E-05 |
| Actinobacillus | Campylobacter | 0.274635 | 2.84E-06 | 3.25E-05 |
| Granulicatella | Veillonella | 0.273761 | 3.07E-06 | 3.44E-05 |
| Streptococcus | Haemophilus | 0.27285 | 3.32E-06 | 3.62E-05 |
| BMI | P/B ratio | 0.272755 | 3.34E-06 | 3.62E-05 |
| (f) Planococcaceae | Veillonella | 0.270121 | 4.19E-06 | 4.45E-05 |
| Actinomyces | Fusobacterium | 0.268817 | 4.68E-06 | 4.88E-05 |
| Tryptophan | Bacteroides | 0.304921 | 5.27E-06 | 5.40E-05 |
| (f) Gemellaceae | Fusobacterium | 0.266633 | 5.62E-06 | 5.58E-05 |
| Actinomyces | Atopobium | 0.266726 | 5.58E-06 | 5.58E-05 |
| BMI | Left Nacc | 0.263659 | 7.20E-06 | 7.03E-05 |
| Veillonella | Lautropia | 0.261342 | 8.72E-06 | 8.37E-05 |
| (f) Neisseriaceae | Lautropia | 0.261084 | 8.91E-06 | 8.41E-05 |
| Haemophilus | Granulicatella | 0.260077 | 9.67E-06 | 8.99E-05 |
| Actinobacillus | Oribacterium | 0.256021 | 1.34E-05 | 0.000121 |
| Veillonella | Atopobium | 0.256063 | 1.34E-05 | 0.000121 |
| Aggregatibacter | Oribacterium | 0.253127 | 1.69E-05 | 0.00015 |
| BMI | Bacteroides | -0.25185 | 1.87E-05 | 0.000161 |
| Actinomyces | Haemophilus | 0.252014 | 1.85E-05 | 0.000161 |
| Aggregatibacter | Fusobacterium | 0.249455 | 2.26E-05 | 0.000192 |
| Granulicatella | Oribacterium | 0.248904 | 2.36E-05 | 0.000198 |
| Oribacterium | Campylobacter | 0.245028 | 3.18E-05 | 0.000263 |
| (f) Ruminococcaceae | Streptococcus | 0.244317 | 3.36E-05 | 0.000274 |
| Fusobacterium | (f) Neisseriaceae | 0.243471 | 3.58E-05 | 0.000288 |
| (f) Planococcaceae | Fusobacterium | 0.241995 | 4.01E-05 | 0.000318 |
| Fusobacterium | Campylobacter | 0.239663 | 4.78E-05 | 0.000374 |
| Lactococcus | Granulicatella | 0.239211 | 4.94E-05 | 0.000382 |
| (f) Gemellaceae | Atopobium | 0.238912 | 5.06E-05 | 0.000386 |
| Aggregatibacter | (f) Gemellaceae | 0.238664 | 5.15E-05 | 0.000388 |
| (o) RF39 | Rothia | 0.237893 | 5.45E-05 | 0.000401 |
| BMI | Tryptophan | -0.27163 | 5.44E-05 | 0.000401 |
| Eubacterium | Granulicatella | 0.235384 | 6.57E-05 | 0.000471 |
| Eubacterium | (f) Planococcaceae | 0.235481 | 6.52E-05 | 0.000471 |
| Lactococcus | (f) Planococcaceae | 0.23418 | 7.17E-05 | 0.000508 |
| Porphyromonas | Oribacterium | 0.232934 | 7.86E-05 | 0.00055 |
| Lactobacillus | Rothia | 0.232074 | 8.36E-05 | 0.000579 |
| (f) Neisseriaceae | Megamonas | 0.230992 | 9.05E-05 | 0.000619 |
| (f) Gemellaceae | Veillonella | 0.230645 | 9.27E-05 | 0.000627 |
| (f) Planococcaceae | Oribacterium | 0.228689 | 0.000107 | 0.000714 |
| Parabacteroides | Granulicatella | 0.227761 | 0.000114 | 0.000754 |
| Streptococcus | Eubacterium | 0.227115 | 0.000119 | 0.000781 |
| Haemophilus | Fusobacterium | 0.226675 | 0.000123 | 0.000786 |
| Megasphaera | Megamonas | 0.226556 | 0.000124 | 0.000786 |
| Streptococcus | Atopobium | 0.226553 | 0.000124 | 0.000786 |
| Aggregatibacter | Rothia | 0.225845 | 0.000131 | 0.000818 |
| Veillonella | Megamonas | 0.224147 | 0.000147 | 0.000885 |
| Prevotella | (f) Victivallaceae | 0.224271 | 0.000146 | 0.000885 |
| (f) Ruminococcaceae | (f) Victivallaceae | 0.224475 | 0.000144 | 0.000885 |
| Bacteroides | Atopobium | 0.22428 | 0.000146 | 0.000885 |
| Streptococcus | Actinobacillus | 0.223597 | 0.000153 | 0.00091 |
| Lactobacillus | Lactococcus | 0.222992 | 0.00016 | 0.00094 |
| (f) Ruminococcaceae | Actinomyces | 0.221693 | 0.000175 | 0.001019 |
| Eubacterium | Lactococcus | 0.221522 | 0.000177 | 0.001021 |
| Fusobacterium | Rothia | 0.220046 | 0.000196 | 0.001119 |
| Granulicatella | Actinobacillus | 0.219862 | 0.000198 | 0.001123 |
| Actinobacillus | Fusobacterium | 0.2192 | 0.000207 | 0.001153 |
| Actinomyces | Lactobacillus | 0.21871 | 0.000214 | 0.001169 |
| Haemophilus | Oribacterium | 0.218599 | 0.000216 | 0.001169 |
| Streptococcus | Oribacterium | 0.218841 | 0.000213 | 0.001169 |
| Fusobacterium | Oribacterium | 0.217976 | 0.000225 | 0.001209 |
| (o) RF39 | Prevotella | 0.217209 | 0.000238 | 0.001262 |
| Veillonella | (f) Neisseriaceae | 0.216267 | 0.000253 | 0.001333 |
| Aggregatibacter | Lautropia | 0.215453 | 0.000267 | 0.001396 |
| BMI | Prevotella | 0.215249 | 0.000271 | 0.001403 |
| Haemophilus | Atopobium | 0.214986 | 0.000276 | 0.001416 |
| Porphyromonas | (f) Neisseriaceae | 0.213115 | 0.000313 | 0.001591 |
| Lactobacillus | (f) Gemellaceae | 0.209709 | 0.000392 | 0.001976 |
| Haemophilus | Lautropia | 0.20941 | 0.0004 | 0.001988 |
| (f) Planococcaceae | Actinobacillus | 0.209362 | 0.000401 | 0.001988 |
| Actinobacillus | Atopobium | 0.20877 | 0.000417 | 0.002049 |
| (f) Gemellaceae | Campylobacter | 0.206605 | 0.00048 | 0.00234 |
| Bacteroides | Granulicatella | 0.20475 | 0.000541 | 0.002615 |
| Eubacterium | (f) Gemellaceae | 0.204325 | 0.000556 | 0.002644 |
| (f) Ruminococcaceae | Rothia | 0.204433 | 0.000552 | 0.002644 |
| Lactobacillus | Oribacterium | 0.204009 | 0.000567 | 0.002677 |
| Tryptophan | Granulicatella | 0.232525 | 0.000589 | 0.002757 |
| (f) Ruminococcaceae | Lactococcus | 0.203222 | 0.000596 | 0.002771 |
| (f) Gemellaceae | Oribacterium | 0.202146 | 0.000638 | 0.002943 |
| (f) Gemellaceae | (f) Neisseriaceae | 0.200225 | 0.00072 | 0.003297 |
| (f) Gemellaceae | Lautropia | 0.199545 | 0.000752 | 0.003414 |
| Actinomyces | Actinobacillus | 0.199127 | 0.000772 | 0.003478 |
| BMI | Fusobacterium | 0.198738 | 0.000791 | 0.003536 |
| Lactococcus | Fusobacterium | 0.197954 | 0.00083 | 0.003625 |
| Fusobacterium | Lautropia | 0.198191 | 0.000818 | 0.003625 |
| Parabacteroides | Actinomyces | 0.197964 | 0.00083 | 0.003625 |
| P/B ratio | (o) RF39 | 0.197865 | 0.000835 | 0.003625 |
| Bacteroides | (f) Victivallaceae | -0.19767 | 0.000845 | 0.003642 |
| Eubacterium | Prevotella | 0.195565 | 0.000962 | 0.004114 |
| Oribacterium | Lautropia | 0.195461 | 0.000968 | 0.004114 |
| Fusobacterium | Porphyromonas | 0.193671 | 0.00108 | 0.004557 |
| cc_115 | (f) Ruminococcaceae | 0.193434 | 0.001096 | 0.00459 |
| Actinomyces | Campylobacter | 0.192797 | 0.001139 | 0.004738 |
| BMI | Oribacterium | 0.192127 | 0.001186 | 0.004899 |
| P/B ratio | Parabacteroides | -0.19156 | 0.001227 | 0.005034 |
| Aggregatibacter | (f) Neisseriaceae | 0.191135 | 0.001259 | 0.00513 |
| Streptococcus | Fusobacterium | 0.190617 | 0.001298 | 0.005226 |
| Lactobacillus | (f) Planococcaceae | 0.190599 | 0.0013 | 0.005226 |
| Atopobium | Oribacterium | 0.190448 | 0.001312 | 0.005238 |
| Atopobium | Campylobacter | 0.189072 | 0.001424 | 0.005648 |
| Haemophilus | (f) Planococcaceae | 0.188781 | 0.001449 | 0.005708 |
| Lactobacillus | Granulicatella | 0.188538 | 0.00147 | 0.005753 |
| BMI | Megamonas | 0.188017 | 0.001516 | 0.005895 |
| Tryptophan | Lautropia | -0.21396 | 0.001602 | 0.006189 |
| (f) Ruminococcaceae | Fusobacterium | -0.18682 | 0.001627 | 0.006245 |
| Lactococcus | Rothia | 0.186177 | 0.001689 | 0.006443 |
| BMI | Campylobacter | 0.185534 | 0.001754 | 0.006647 |
| Prevotella | Megasphaera | 0.185237 | 0.001785 | 0.00672 |
| (f) Planococcaceae | Porphyromonas | 0.184457 | 0.001867 | 0.006988 |
| Streptococcus | Campylobacter | 0.180826 | 0.002302 | 0.008559 |
| Veillonella | Porphyromonas | 0.180685 | 0.00232 | 0.008575 |
| Atopobium | Lautropia | 0.180237 | 0.00238 | 0.008742 |
| (o) RF39 | Lactobacillus | 0.179819 | 0.002437 | 0.008897 |
| P/B ratio | Megasphaera | 0.174853 | 0.003219 | 0.01168 |
| Porphyromonas | Lautropia | 0.174542 | 0.003275 | 0.011811 |
| Atopobium | Megamonas | 0.173242 | 0.003518 | 0.012611 |
| Actinomyces | Lautropia | 0.168809 | 0.004474 | 0.015942 |
| Rothia | Oribacterium | 0.1684 | 0.004573 | 0.016197 |
| Actinomyces | Aggregatibacter | 0.168243 | 0.004612 | 0.016238 |
| Rothia | (f) Neisseriaceae | 0.167055 | 0.004913 | 0.017196 |
| Lactococcus | Oribacterium | 0.165283 | 0.005396 | 0.018775 |
| Rothia | Campylobacter | 0.164682 | 0.005569 | 0.019264 |
| (f) Ruminococcaceae | Parabacteroides | 0.163286 | 0.00599 | 0.020477 |
| Aggregatibacter | Porphyromonas | 0.163182 | 0.006023 | 0.020477 |
| P/B ratio | Megamonas | 0.163303 | 0.005985 | 0.020477 |
| Actinomyces | Megamonas | 0.162694 | 0.006177 | 0.020883 |
| (o) RF39 | Granulicatella | 0.162239 | 0.006325 | 0.02126 |
| Bacteroides | Streptococcus | 0.160431 | 0.006942 | 0.023204 |
| Haemophilus | (f) Neisseriaceae | 0.159583 | 0.007249 | 0.024096 |
| BMI | (f) Victivallaceae | 0.158647 | 0.007603 | 0.025131 |
| P/B ratio | Eubacterium | 0.158287 | 0.007743 | 0.025453 |
| (f) Neisseriaceae | Campylobacter | 0.156805 | 0.008344 | 0.027101 |
| Eubacterium | Rothia | 0.156716 | 0.008381 | 0.027101 |
| Rothia | Lautropia | 0.156757 | 0.008364 | 0.027101 |
| Tryptophan | Streptococcus | 0.178803 | 0.008597 | 0.027649 |
| Aggregatibacter | Atopobium | 0.155979 | 0.008696 | 0.027818 |
| Prevotella | Fusobacterium | 0.155542 | 0.008888 | 0.02813 |
| Lactobacillus | Prevotella | 0.155607 | 0.008859 | 0.02813 |
| Actinobacillus | Lautropia | 0.155411 | 0.008946 | 0.028164 |
| Tryptophan | (f) Planococcaceae | 0.175426 | 0.009958 | 0.031185 |
| Atopobium | (f) Victivallaceae | 0.153031 | 0.010065 | 0.031354 |
| cc_115 | Rothia | 0.152581 | 0.01029 | 0.031887 |
| Lactobacillus | Actinobacillus | 0.151078 | 0.011074 | 0.03414 |
| (f) Planococcaceae | Aggregatibacter | 0.149924 | 0.011711 | 0.035918 |
| Atopobium | Fusobacterium | 0.149801 | 0.011781 | 0.035947 |
| BMI | Lautropia | 0.149365 | 0.012031 | 0.036524 |
| Parabacteroides | (f) Gemellaceae | 0.148754 | 0.01239 | 0.037232 |
| Parabacteroides | Lactococcus | 0.148822 | 0.01235 | 0.037232 |
| BMI | (f) Gemellaceae | 0.148397 | 0.012604 | 0.037685 |
| Megamonas | Lautropia | 0.146988 | 0.01348 | 0.040104 |
| cc_115 | Lactococcus | 0.146085 | 0.01407 | 0.041649 |
| Granulicatella | Campylobacter | 0.145769 | 0.014281 | 0.042067 |
| Megasphaera | Lautropia | 0.145146 | 0.014706 | 0.043105 |
| Granulicatella | Porphyromonas | 0.144401 | 0.01523 | 0.044421 |
| Granulicatella | Fusobacterium | 0.143994 | 0.015523 | 0.044962 |
| Tryptophan | (f) Neisseriaceae | -0.1648 | 0.015567 | 0.044962 |
| P/B ratio | Tryptophan | -0.16427 | 0.015904 | 0.045716 |
| (f) Ruminococcaceae | Granulicatella | 0.14312 | 0.016168 | 0.046249 |
| (f) Ruminococcaceae | Campylobacter | -0.14067 | 0.018099 | 0.051525 |
| Granulicatella | Megasphaera | 0.14055 | 0.018202 | 0.051571 |
| Lactobacillus | Porphyromonas | 0.139662 | 0.018955 | 0.05345 |
| Bacteroides | Actinomyces | 0.139258 | 0.019306 | 0.054184 |
| Actinobacillus | Porphyromonas | 0.138822 | 0.019692 | 0.055009 |
| Streptococcus | Aggregatibacter | 0.138094 | 0.020351 | 0.056583 |
| Lactobacillus | Veillonella | 0.137905 | 0.020525 | 0.056801 |
| Parabacteroides | Eubacterium | 0.137698 | 0.020717 | 0.057068 |
| Prevotella | Megamonas | 0.137487 | 0.020914 | 0.057345 |
| Parabacteroides | Rothia | 0.137219 | 0.021168 | 0.057775 |
| (f) Victivallaceae | Rothia | 0.136459 | 0.0219 | 0.059501 |
| Megasphaera | Campylobacter | 0.136122 | 0.022232 | 0.059857 |
| Oribacterium | Megamonas | 0.136124 | 0.02223 | 0.059857 |
| Parabacteroides | Streptococcus | 0.135951 | 0.022402 | 0.060041 |
| Lactobacillus | Atopobium | 0.135453 | 0.022903 | 0.06111 |
| Bacteroides | Porphyromonas | 0.135328 | 0.023032 | 0.061178 |
| cc_115 | Actinomyces | 0.135109 | 0.023256 | 0.061499 |
| Tryptophan | Campylobacter | -0.15445 | 0.023512 | 0.0619 |
| BMI | Veillonella | 0.13443 | 0.023965 | 0.062816 |
| P/B ratio | Left Nacc | 0.13332 | 0.025163 | 0.065667 |
| Tryptophan | Actinomyces | 0.152077 | 0.025755 | 0.066919 |
| Lactobacillus | (f) Victivallaceae | 0.132485 | 0.026099 | 0.067518 |
| Atopobium | Rothia | 0.131735 | 0.026965 | 0.069455 |
| (f) Ruminococcaceae | Aggregatibacter | -0.13161 | 0.027112 | 0.069533 |
| Tryptophan | (f) Gemellaceae | 0.150498 | 0.027353 | 0.069849 |
| Brainstem | Oribacterium | -0.13121 | 0.027583 | 0.070137 |
| BMI | (f) Neisseriaceae | 0.128536 | 0.030939 | 0.078002 |
| Streptococcus | Porphyromonas | 0.127931 | 0.031744 | 0.079694 |
| Prevotella | (f) Gemellaceae | 0.127815 | 0.031901 | 0.079752 |
| Granulicatella | Prevotella | 0.126243 | 0.034087 | 0.084861 |
| Atopobium | Porphyromonas | 0.126098 | 0.034294 | 0.085022 |
| (f) Ruminococcaceae | (f) Neisseriaceae | -0.1259 | 0.034579 | 0.085371 |
| Streptococcus | Megasphaera | 0.125246 | 0.035537 | 0.087015 |
| Brainstem | (f) Gemellaceae | -0.12526 | 0.03552 | 0.087015 |
| Brainstem | Actinomyces | -0.12514 | 0.035698 | 0.087051 |
| Lactococcus | Veillonella | 0.12443 | 0.036763 | 0.089282 |
| Brainstem | Fusobacterium | -0.12407 | 0.037315 | 0.090253 |
| Haemophilus | Lactococcus | 0.123549 | 0.038128 | 0.091847 |
| Actinobacillus | (f) Neisseriaceae | 0.12319 | 0.038697 | 0.092841 |
| Haemophilus | Lactobacillus | 0.121845 | 0.040888 | 0.097703 |
| (f) Gemellaceae | Megasphaera | 0.120697 | 0.042842 | 0.097031 |
| BMI | Eubacterium | 0.119728 | 0.044551 | 0.097703 |
| cc_115 | (f) Victivallaceae | 0.119417 | 0.04511 | 0.097703 |
| Lactobacillus | Eubacterium | 0.118461 | 0.046871 | 0.09782 |
| Campylobacter | Megamonas | 0.118464 | 0.046865 | 0.09784 |
| (f) Gemellaceae | Porphyromonas | 0.118096 | 0.047558 | 0.098503 |
| Bacteroides | Actinobacillus | 0.117971 | 0.047796 | 0.09785 |
| Lactococcus | Actinobacillus | 0.117939 | 0.047856 | 0.09882 |
| Left Nacc | Bacteroides | -0.11833 | 0.047121 | 0.09883 |
| Tryptophan | Parabacteroides | 0.13513 | 0.047825 | 0.09891 |
| Granulicatella | Aggregatibacter | 0.116943 | 0.049783 | 0.099603 |
| Left Nacc | Eubacterium | 0.116652 | 0.04979 | 0.099703 |

**Table S3:** Strength of each spearman correlation seen in the analysis used to generate the circos plot (including, nonsignificant relationships) (i.e. Fig.4)

***Supplemental Figures***


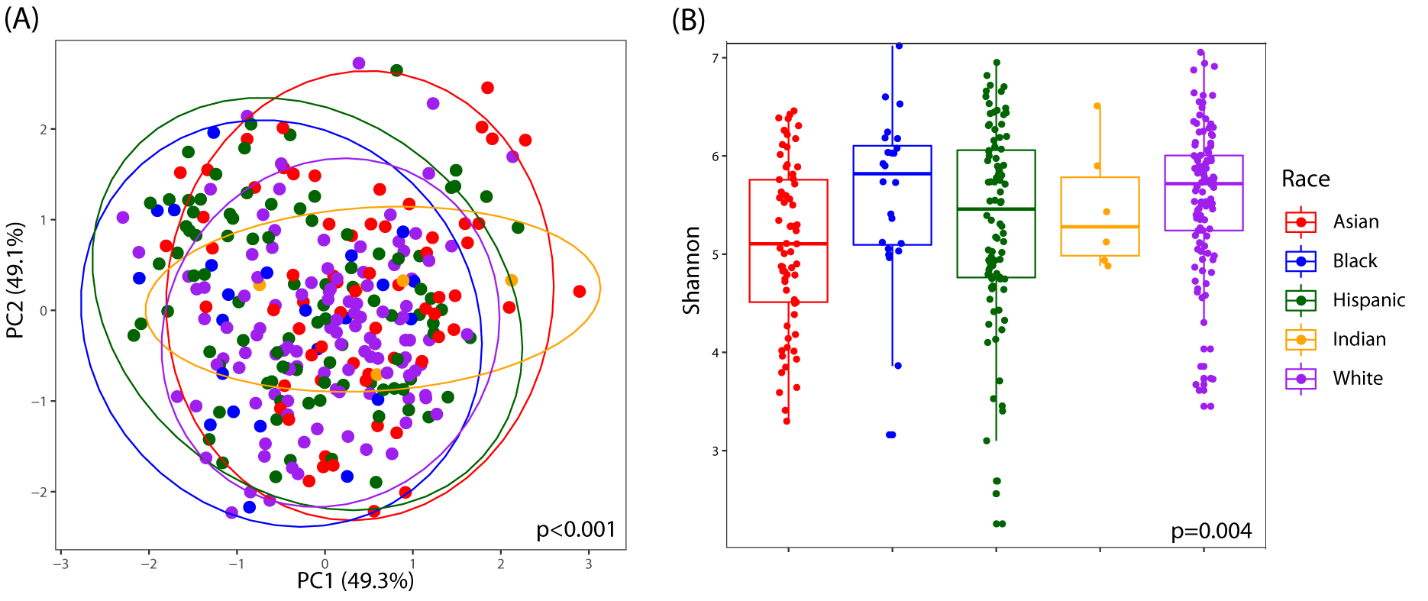


**Fig. S1: Alpha & beta diversity varies by race.** (A) Principal coordinate analysis plot of the microbiome colored by race, encircled by 99% confidence interval ellipses. (B) Box plots representing alpha diversity using the Shannon index (measure of richness and evenness) grouped by race.


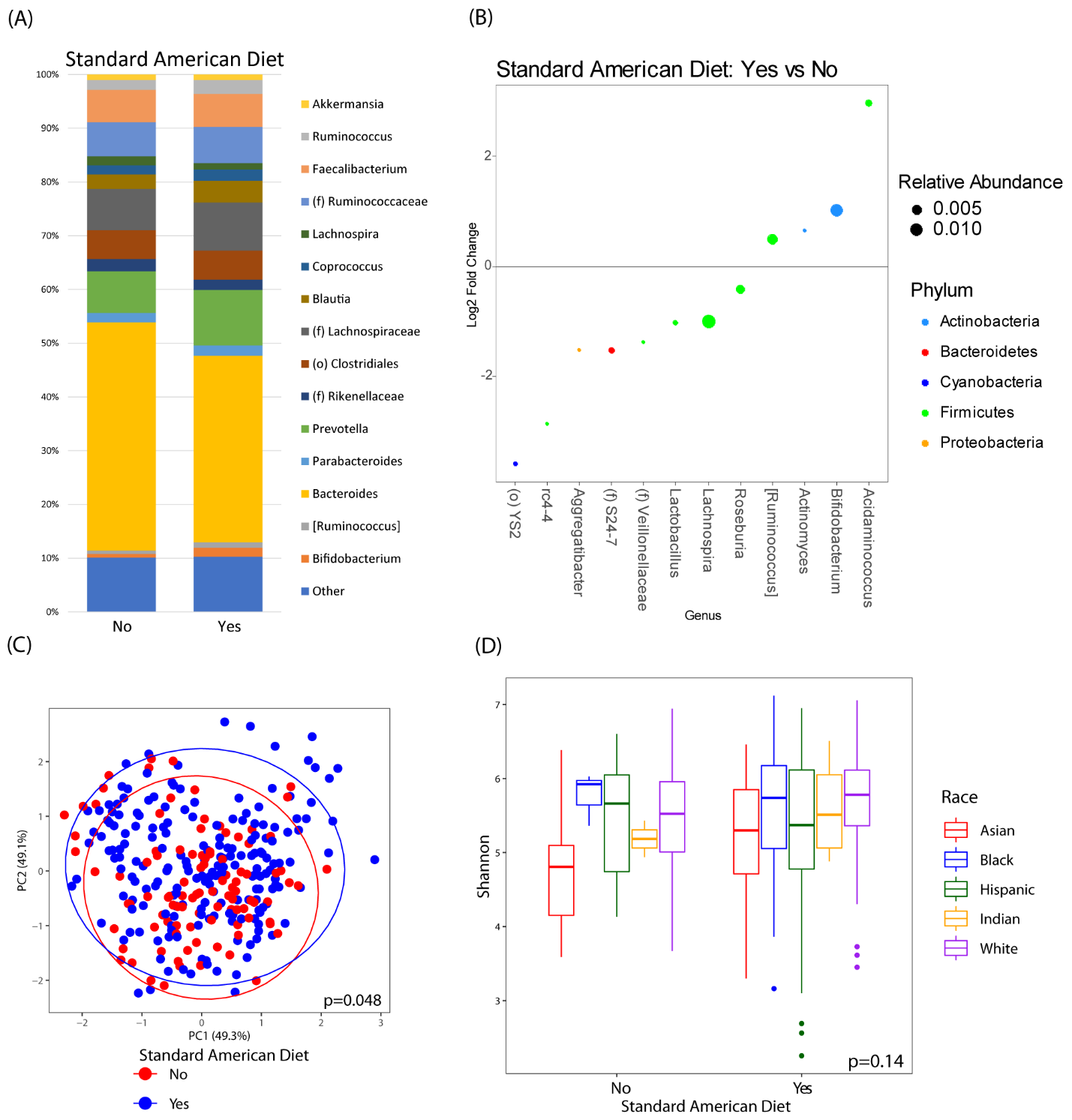


**Fig. S2: Microbiome diversity and composition varies by Standard American Diet.** (A) Taxonomic summary plots showing relative abundance of all genera (minimum of 1% relative abundance) by diet (Standard American Diet vs. non- Standard American Diet)**. (**B) Log2 fold changes for genera with differential abundance between those on Standard American Diet vs. non-Standard American Diet in DESeq2 models adjusting for race and obesity (q<0.05). (C) Principal coordinate analysis plot of the microbiome based on diet (Standard American Diet vs. non-Standard American Diet) encircled by 99% confidence interval ellipses**. (**D) Box-plot of microbial diversity by Shannon index (measure of richness and evenness) grouped by diet and stratified by race


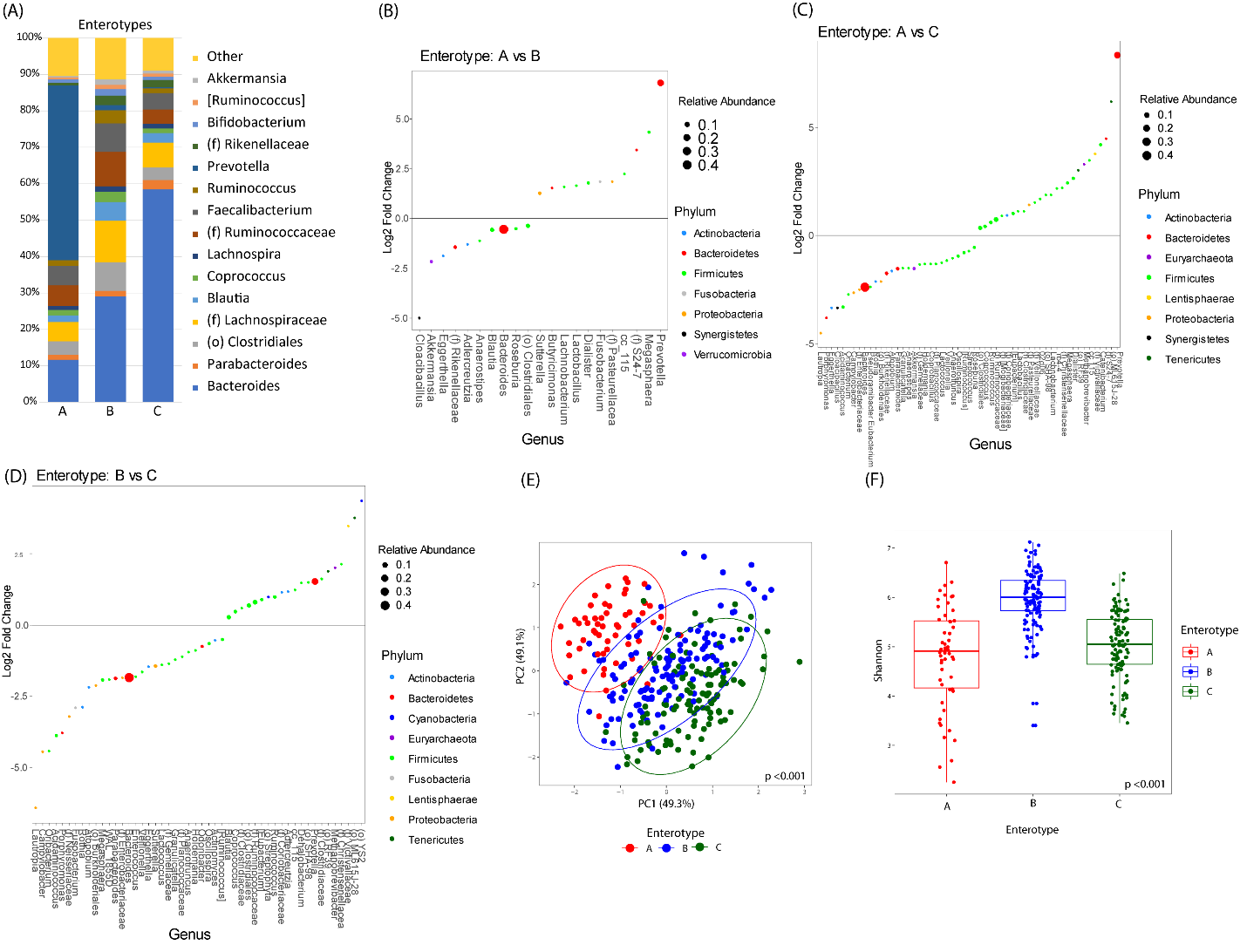


**Fig. S3: Enterotype-based differences in microbiome diversity and composition that correlates with Obesity.** A) Taxonomic profiles showing relative abundance of all genera across the three enterotypes. DESeq2 differential abundance analysis comparing: B) enterotype A to B, (C) enterotype A to C, and **(**D) enterotype B to C adjusting for race, obesity, and diet**.** E) Principal coordinate analysis plot of beta diversity between three enterotypes. F) Shannon index box-plots of alpha diversity between three enterotypes
